# Risk factors linked to gastric intestinal metaplasia in adults from a private clinic in the Dominican Republic

**DOI:** 10.1016/j.igie.2025.12.010

**Published:** 2025-12-27

**Authors:** Pablo Socias-Pappaterra, Yousef S. Ebrahim Ibrahim, Vivian Ho Sang, Beatriz Moquete Grullon, Ramon Romano, Fernando Contreras

**Affiliations:** 1 Department of Knowledge Management and Epidemiology, Centros de Diagnóstico y Medicina Avanzada y de Conferencias Médicas y Telemedicina (CEDIMAT), Santo Domingo, Dominican Republic; 2 Centro de Gastroenterología Avanzada, Santo Domingo, Dominican Republic

## Abstract

**Background and Aims:**

Gastric intestinal metaplasia (GIM) significantly increases the risk of developing future gastric cancer by 6-fold. This study aimed to identify the most common risk factors associated with GIM in adults who underwent EGD at a private clinic in the Dominican Republic.

**Methods:**

This retrospective cohort study examined the risk factors of GIM in adults who underwent esophagogastroduodenoscopy (EGD) at a private clinic in the Dominican Republic in 2024. Indications included dyspepsia, gastroesophageal reflux disease (GERD), surveillance of premalignant lesions, combined lower and upper gastrointestinal screening, and alarm symptoms. All procedures followed the Sydney biopsy protocol, with additional biopsy specimens from areas concerning for GIM. Experienced pathologists reviewed biopsy specimens and diagnosed GIM histologically. Patient data and biopsy results were obtained from electronic medical records, and logistic regression identified independent risk factors for GIM.

**Results:**

Of the 781 patients, 14.6% had GIM. Multivariate odds ratio (MultivOR) analysis indicated that current *Helicobacter pylori* (*H pylori*) infection (MultivOR, 3.51; 95% confidence interval [CI], 2.01-6.10), past *H pylori* infection (MultivOR, 2.61; 95% CI, 1.63-4.16), and age (MultivOR, 1.06; 95% CI, 1.04-1.08) were linked to an increased risk of GIM. Other factors showed no significant association.

**Conclusions:**

Current and past *H pylori* infections and age increase GIM risk for both extensive and limited GIM in adults undergoing EGD at a Dominican clinic. Further studies are needed to evaluate their relevance for gastric cancer screening strategies.

## Background

Gastric intestinal metaplasia (GIM) is a change from specialized gastric cells to intestinal-type cells, serving as a precursor to gastric cancer with at least a 6-fold increased risk of gastric adenocarcinoma.^1^ It ranks as the third-leading cause of cancer-related deaths worldwide.^1^ GIM is categorized as complete (type 1) or incomplete (types 2 and 3), with type 3 having a higher risk for dysplasia and intestinal-type gastric cancer. The condition is more prevalent among Hispanics and East Asians.^1^ Risk factors include *Helicobacter pylori* (*H pylori*) infection, high body mass index (BMI), age over 50 years, low socioeconomic status, smoking, alcohol, reheated foods, salt intake, male sex, country of birth, race, and family history of gastric cancer.^2^^,^^3^

*H pylori* infection is the most established risk factor, causing chronic inflammation that can lead to chronic gastritis, atrophy, metaplasia, dysplasia, and adenocarcinoma.^1^ Moreover, elevated BMI is linked to incomplete GIM progression, which is 3 times more likely to advance to gastric cancer than complete GIM.^2^ Other risk factors can further exacerbate this progression. Managing *H pylori* infection, patients' BMI, and related risk factors can limit GIM severity, decrease gastric cancer incidence, and improve patient outcomes.^2^

*H pylori* infection and GIM prevalence in the Dominican Republic are poorly understood and vary between private and public settings. A University of Miami Hospital (Miami, Fla, USA) study (2011-2021) reported *H pylori* infection in 18.4% of Dominican patients undergoing esophagogastroduodenoscopy (EGD),^3^ whereas regional prevalence in Latin America and the Caribbean reached 57.6% across all ages.^4^ Although multiple risk factors for GIM are recognized in the Dominican population, their associations remain poorly characterized.

Prior studies include few Dominican patients, limiting generalizability, and most focus primarily on *H pylori* infection while omitting other established risk factors, often because of incomplete clinical data.^3^ Moreover, only a small number of studies have stratified risk factors according to the extension of GIM, to our knowledge, creating uncertainty about associations with disease progression in the Dominican population. These limitations emphasize the need for studies with larger Dominican cohorts, comprehensive risk factor assessment, and stratification of GIM extension to define disease patterns.

In highly prevalent countries, screening EGD can be performed to detect metaplasia, dysplasia, and early gastric cancer (EGC). Routine EGD is recommended depending on the histologic risk for malignant transformation and surgical or endoscopic resection for highly concerning lesions.^1^^,^^5^ In some high-risk countries such as Japan, screening protocols have been implemented, which have helped detect up to 70% of EGC. This can boost 5-year survival rates from 10% to 30% to 70% to 90%.^6^^,^^7^

In high-risk countries such as China, the EGC detection rate is under 10%, partly because of the large population and uneven distribution of medical resources. Similar limitations exist in the Dominican Republic, where data on the prevalence of GIM and its risk factors are lacking. Identifying these associations is essential for early detection and prevention in high-risk populations. Such evidence could help develop targeted screening protocols, similar to those in Japan.^7^

This observational study identified common risk factors for GIM in adults who underwent EGD at a private gastroenterology clinic in the Dominican Republic in 2024. The study also determined the prevalence of GIM, characterized the most prevalent GIM subtype, and located anatomical regions of the stomach with higher frequencies of GIM. In addition, it compared risk factors based on the extent of GIM within the gastric mucosa.

## Methods

### Study design

This single-center retrospective cohort study was conducted to identify risk factors among patients who underwent EGD at the Centro de Gastroenterología Avanzada (CGA) in Santo Domingo, Dominican Republic, in 2024 and to evaluate the relationship between GIM biopsy findings and the presence of these risk factors. The researchers adhered to the STrengthening the Reporting of OBservational studies in Epidemiology guidelines to ensure that the results were reported with clarity and coherence.

### Setting

The study was performed at CGA, a gastroenterology-focused clinic that provides outpatient consultations, emergency care, and diagnostic and therapeutic endoscopic procedures to a primarily urban, high-income population. For study inclusion, electronic medical records were reviewed to identify all adults who underwent EGD between January and December 2024.

### Participants

A total of 979 adults who underwent EGD at the CGA in 2024 were initially recruited for this study. Indications for EGD included dyspepsia, gastroesophageal reflux disease (GERD), surveillance of premalignant lesions, alarm symptoms, and combined lower and upper gastrointestinal screening. Combined screening was offered primarily to adults over 45 years undergoing colonoscopy as part of their preventative checkup.

Inclusion criteria included adults who underwent EGD during the study period. Exclusion criteria were incomplete medical records, absence of gastric biopsies, and age under 18 years. Patients with multiple EGDs in 2024 had only the first reviewed; subsequent endoscopies were excluded. Selection was via nonprobabilistic convenience sampling. Patients who underwent EGD for the treatment of gastric cancer, management of peptic ulcers, or those who had postsurgical anatomy were excluded.

### Outcomes

The study primarily aimed to establish the correlation between GIM presence and risk factors: *H pylori* infection (past/current), age, sex, alcohol/smoking history, family history of gastric cancer, BMI, proton pump inhibitors (PPIs), nonsteroidal anti-inflammatory drugs (NSAIDs), GERD, and dyspepsia. Secondary outcomes included the prevalence of GIM and its subtypes among clinic patients, gastric location proportions, predicted GIM probability by age and *H pylori* status, and comparison of limited versus extensive GIM associations.

### Variables

The protocol was designed to gather sociodemographic, clinical, and biopsy results–related variables. Sociodemographic variables included age and sex, whereas clinical variables included BMI, alcohol and smoking history, PPIs, NSAIDs, GERD, dyspepsia, *H pylori* (current and past), and presence, type, and location of GIM.

Patients with histologic confirmation of GIM were categorized as having limited or extensive GIM, depending on the anatomical extent of the lesions. Limited GIM was defined as involvement restricted to the distal stomach (antrum, incisura, and/or prepyloric region) without corpus or fundus involvement. Extensive GIM was defined as involvement of the corpus and/or fundus, either alone or in combination with distal sites.

### Routine protocol for EGD examination

Endoscopic examinations were conducted by 3 experienced endoscopists (F.C., N.P.S., M.A.) using Fujifilm's ELUXEO Ultra endoscopes (Fujifilm, Tokyo, Japan). To optimize mucosal visualization, all patients received simethicone and N-acetylcysteine 30 minutes before the procedure. Residual foam was minimized using water infused with simethicone during the examination.

The mucosa was assessed in a standardized sequence, initially with white light, followed by linked-color imaging with and without magnification. Then, blue-light imaging was used with and without magnification. Each procedure lasted at least 7 minutes from scope insertion to withdrawal, and all endoscopists adhered to the same unified protocol.

Routine biopsy specimens were taken in the antrum, incisura, and corpus, following guidelines from the Sydney protocol, with additional targeted biopsy specimens from endoscopically visualized areas concerning for GIM. Clinical pathologists performed histopathologic evaluation of each sample. *H pylori* infection was detected using the special Giemsa stain.

To classify GIM, pathologists used the following criteria for histologic diagnoses: for complete GIM, they observed the presence of straight crypt architecture, well-developed goblet cells, eosinophilic enterocytes with a brush border, and Paneth cells at the crypt base. For incomplete GIM, they observed a more irregular architecture, greater variation in goblet cell size, and the presence of intermediate columnar cells in various stages of differentiation containing intracytoplasmic mucin.

### Surveillance protocol at CGA

At CGA, patients are routinely followed based on their EGD results. Those with extensive or incomplete GIM, family history of gastric cancer, persistent *H pylori* infection, an Operative Link of Gastritis Assessment stage III to IV, or an endoscopic grading of GIM score of 5 are usually placed in a surveillance protocol with repeat EGD in 1 to 3 years. For moderate-risk patients, their next EGD is offered as part of their preventative checkup, typically during a colonoscopy. This approach mimics the management of epithelial precancerous conditions and lesions in the stomach III recommendations, with CGA offering additional opportunistic endoscopy to moderate-risk individuals as part of preventative care.^8^

### Data sources and data collection

Ethical standards were followed while examining electronic medical records. Information was gathered on the basis of the history and physical examination notes from the database, EGD findings, and biopsy results. Deidentified data included gastric biopsy results and their respective anatomical locations, presence of *H pylori*, height and weight to calculate BMI, age, sex, social history, family history, and other sociodemographic variables. Data were gathered using KoboToolBox (Kobo Organization, Cambridge, Mass, USA), whereas statistical analysis was completed with the Stata/BE 18 software (College Station, Tex, USA). Institutional Review Board (IRB) approval from the CGA and Comité de Ética de Investigación de Centros de Diagnóstico y Medicina Avanzada y de Conferencias Médicas y Telemedicina was obtained to guarantee systematic and ethical data acquisition, and nondisclosure agreements were signed (IRB14368-CEI-067).

### Bias

The study contemplated that confounding and selection bias were the main obstacles to its external and internal validity. For selection bias, although the sample size proved robust enough for the proposed analysis, the sampling methodology did not include probabilistic methods. For confounding factors, during the proposal phase, an analysis of possible confounders revealed the necessity of undertaking regression analysis to control for predictors and potential confounders, including age, sex, and current *H pylori* infection. However, an unknown degree of selection bias and confounding effect can be expected even with the study's sample size and correlation analysis. Data on racial background and country of origin were not found in the medical records; hence, they were not collected.

### Sample size

The initial analysis for logistic regression included a sample of 118 patients. We anticipated a medium effect size of 0.15, with a β of 20% for an 80% statistical power, 10 predictors, and an α of 5%. To address the reduced external validity caused by selection bias, all patients were considered after 198 were excluded, leaving 781 as the final sample size, which met the sample analysis requirements (Fig. 1).

**Figure 1:**
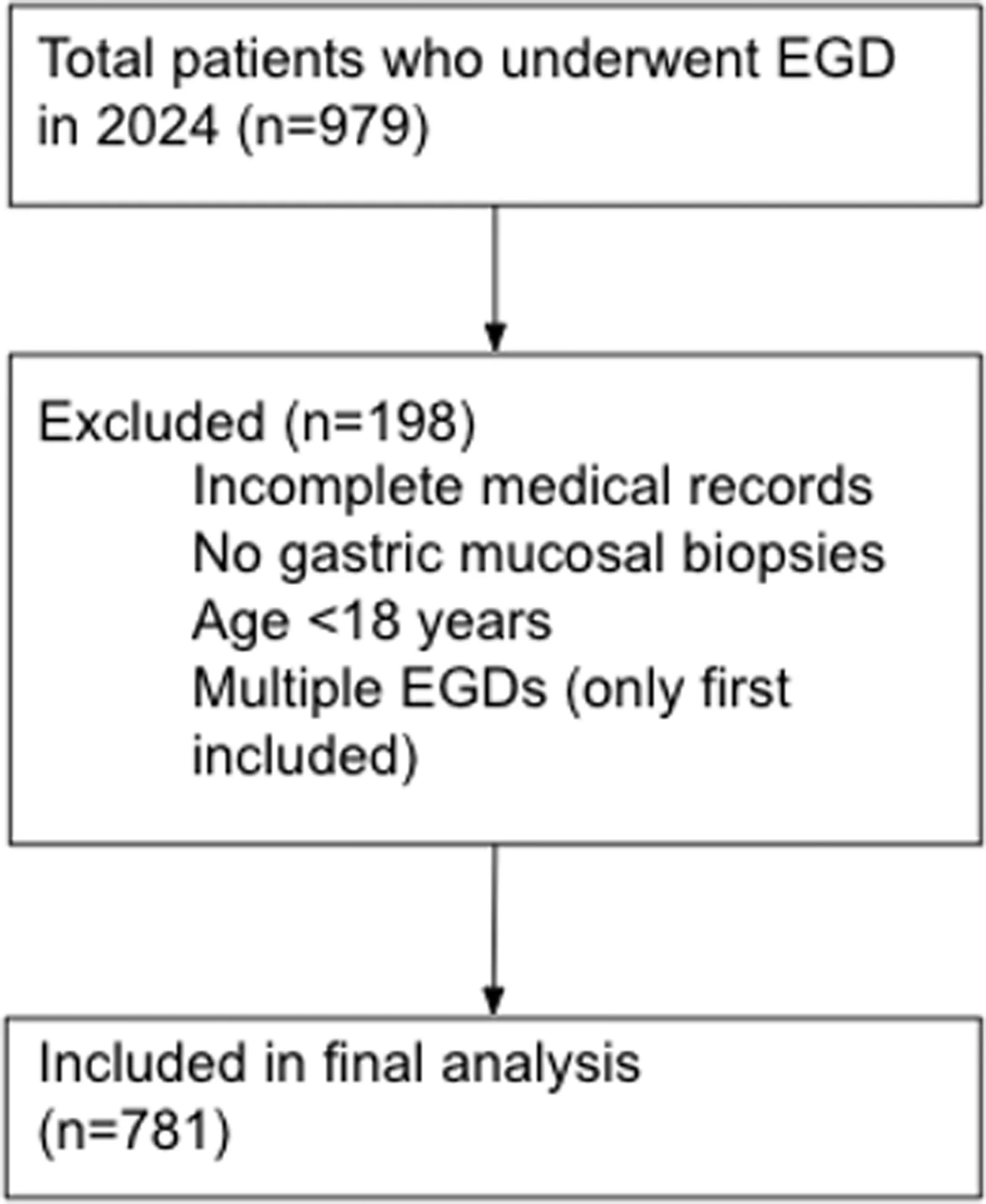
Participants flow diagram. *EGD*, esophagogastroduodenoscopy.

### Statistical analysis

Quantitative and qualitative variables were summarized using mean, standard deviation (SD), and absolute and relative frequencies. For the primary outcome, logistic regression was implemented to obtain the odds ratio (OR) for the risk factors hypothesized to be associated with the presence of GIM and the extension of the lesions. Statistical analysis used 80% power and *P* < .05 for statistical significance. All descriptive and inferential analyses were performed with the Stata software.

After univariate models were performed, multivariable modeling occurred between different levels to study the other GIM risk factors and adjust for possible confounding effects. Univariate models were adjusted for the presence of *H pylori*, age, and sex to determine whether the associations remained.

## Results

A total of 979 patients were initially recruited for this study. After exclusion, 781 patients were ultimately included in this cohort, of whom 114 (14.6%) had GIM (Table 1). Gender-wise, 65 (57%) were male, and 343 (51.4%) were female. Patients with GIM were older: mean age 62.4 years (SD) (10.9) versus 53.9 years (13.1). Among patients with GIM, 25 (21.9%) had *H pylori*, compared with 55 (8.3%) among patients without GIM. In addition, 42 patients (36.8%) with GIM and 95 patients (14.2%) without GIM had previous *H pylori* infections (Table 1). The antrum was the most frequent site of GIM, accounting for 59 (35.5%), and 109 cases (95.6%) of GIM were of the complete subtype (Table 1).

**Table 1:** Characteristics of GIM and sociodemographic characteristics of patients with or without GIM

Variables	GIM present (n = 114)	GIM not present (n = 667)
Age (years), mean (SD)∗	62.4 (10.9)	53.9 (13.1)
Sex, n (%)		
Female	49 (43.0)	343 (51.4)
Male	65 (57.0)	324 (48.6)
BMI, mean (SD)	27.3 (10.9)	26.7 (13.1)
Alcohol history, n (%)		
Never	36 (39.1)	185 (33.0)
Former	2 (2.0)	11 (2.0)
Current	54 (58.7)	364 (65.0)
Smoking history, n (%)		
Never	65 (69.9)	396 (70.2)
Former	16 (17.2)	95 (16.8)
Current	12 (12.9)	73 (13.0)
Current *H pylori* infection, n (%)∗		
Not present	89 (78.1)	612 (91.8)
Present	25 (21.9)	55 (8.3)
Past *H pylori* infection, n (%)∗		
Not present	71 (62.8)	571 (85.7)
Present	42 (37.2)	95 (14.3)
Location of GIM, n = 166 (%)		
Antrum	59 (35.5)	N/A
Corpus	43 (25.9)	N/A
Incisura	27 (16.3)	N/A
Prepyloric	26 (15.7)	N/A
Fundus	11 (6.6)	N/A
Types of GIM, n = 114 (%)		
Complete	109 (95.6)	N/A
Incomplete	5 (4.4)	N/A

*BMI*, Body mass index; *GIM*, gastric intestinal metaplasia; *H pylori*, *Helicobacter pylori*; *N/A*, not available; *SD*, standard deviation.

∗ Statistically significant differences between GIM and non-GIM groups.

Table 2 presents logistic regressions of risk factors associated with GIM. Univariate OR and multivariate OR (MultivOR) adjusted for *H pylori*, age, and sex were analyzed. MultivOR revealed significant associations: current *H pylori* infection (MultivOR, 3.51; 95% CI, 2.01-6.10), past *H pylori* infection (MultivOR, 2.61; 95% CI, 1.63-4.16), and age (MultivOR, 1.06; 95% CI, 1.04-1.08) increased GIM risk. Other risk factors showed no significant associations (Table 2).

**Table 2:** Association between sociodemographic, behavioral, and clinical risk factors with the presence of GIM

Variables	Univariate OR (95% CI)	*P* value	Multivariate OR (95% CI)	*P* value
Current *H pylori* infection	3.13 (1.85-5.27)	<.01	3.51 (2.01-6.10)	<.01
Past *H pylori* infection	3.56 (2.30-5.52)	<.01	2.61 (1.63-4.16)	<.01
Age (years)	1.06 (1.04-1.08)	<.01	1.06 (1.04-1.08)	<.01
Female sex	0.71 (0.48-1.06)	.10	0.71 (0.47-1.09)	.12
BMI	1.03 (0.98-1.08)	.31	1.03 (0.97-1.09)	.30
Family history of gastric cancer	1.70 (0.62-4.69)	.30	2.23 (0.76-6.53)	.14
Alcohol history				
Former	0.93 (0.20-4.39)	.93	0.82 (0.16-4.05)	.80
Current	0.76 (0.48-1.20)	.25	0.86 (0.53-1.41)	.56
Smoking history				
Former	1.03 (0.57-1.85)	.93	0.83 (0.44-1.54)	.55
Current	1.00 (0.52-1.95)	.99	1.13 (0.56-2.29)	.55
PPI use	1.16 (0.73-1.84)	.54	1.21 (0.74-1.99)	.46
NSAID use	1.06 (0.61-1.83)	.84	1.05 (0.59-1.86)	.88
GERD	0.75 (0.49-1.17)	.21	1.01 (0.64-1.61)	.96
Dyspepsia	0.65 (0.37-1.13)	.13	0.84 (0.46-1.51)	.56

Multivariate OR adjusted for *H pylori*, age, and sex.

*BMI*, Body mass index; *CI,* confidence interval; *GERD,* gastroesophageal reflux disease; *GIM*, gastric intestinal metaplasia; *H pylori*, *Helicobacter pylori*; *NSAID*, nonsteroidal anti-inflammatory drug; *OR*, odds ratio; *PPI*, proton pump inhibitor.

Among patients with GIM, limited GIM represented 67 (58.8%) and extensive GIM 47 (41.2%). Both subgroups demonstrated significant associations with current and past *H pylori* infection and age. Other factors were not significantly associated with the extent of GIM (Table 3).

**Table 3:** Association between sociodemographic, behavioral, and clinical risk factors with the presence of extensive and limited GIM

Variables	Extensive GIM Univariate OR (95% CI)	*P* value	Limited GIM Univariate OR (95% CI)	*P* value
Current *H pylori* infection	2.56 (1.22-5.38)	.01	2.88 (1.54-5.40)	<.01
Past *H pylori* infection	3.89 (2.11-7.17)	<.01	2.60 (1.50-4.50)	<.01
Age	1.06 (1.04-1.09)	<.01	1.05 (1.02-1.07)	<.01
Female sex	0.95 (0.53-1.71)	.86	0.60 (0.36-1.01)	.06
BMI	1.02 (0.94-1.10)	.64	1.03 (0.97-1.09)	.38
Family history of gastric cancer	0.74 (0.10-5.63)	.77	2.34 (0.77-7.10)	.13
Alcohol history				
Former	1 (empty)		2.33 (0.47-11.43)	.30
Current	0.43 (0.22-0.83)	.01	1.24 (0.68-2.29)	.48
Smoking history				
Former	1.04 (0.44-2.45)	.93	1.01 (0.47-2.16)	.98
Current	0.37 (0.09-1.59)	.18	1.53 (0.73-3.20)	.26
PPI use	0.69 (0.32-1.50)	.35	1.56 (0.89-2.71)	.12
NSAID use	1.15 (0.52-2.52)	.73	0.99 (0.49-2.00)	.98
GERD	0.60 (0.30-1.19)	.14	0.91 (0.53-1.56)	.73
Dyspepsia	0.48 (0.19-1.24)	.13	0.81 (0.41-1.58)	.54

*BMI*, Body mass index; *CI,* confidence interval; *GERD, gastroesophageal reflux disease;GIM*, gastric intestinal metaplasia; *H pylori*, *Helicobacter pylori*; *NSAID*, nonsteroidal anti-inflammatory drug; *OR*, odds ratio; *PPI*, proton pump inhibitor.

Figure 2 predicts the GIM risk, adjusted for age and *H pylori.* It shows a higher probability of GIM after age adjustment. The probability significantly increases in patients with *H pylori* compared with those without (Fig. 2).

**Figure 2:**
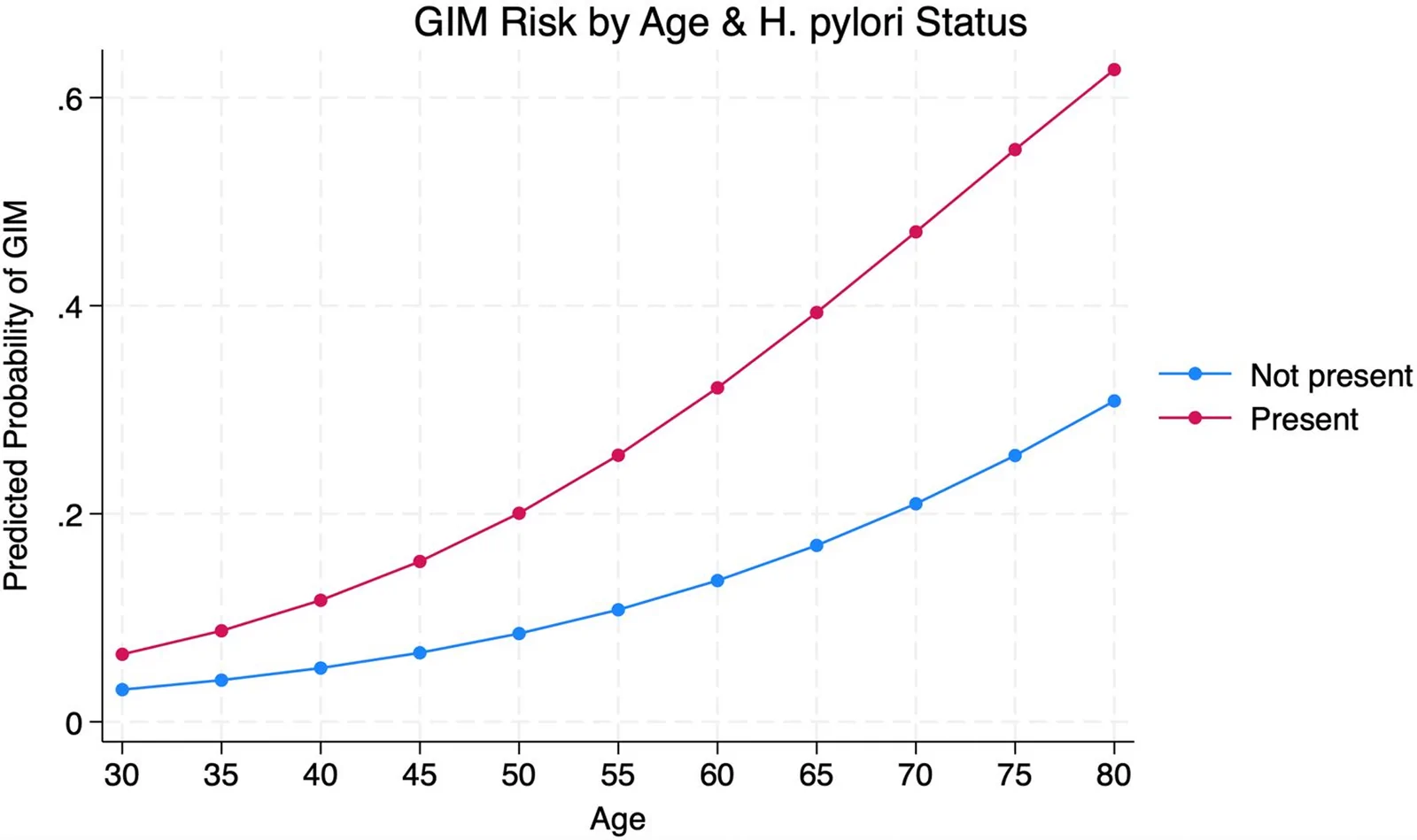
Predicted probability of gastric intestinal metaplasia (GIM) adjusted for age and current *Helicobacter pylori* (*H pylori*) infection. *Not present*, current *H pylori* infection not present; *Present*, current *H pylori* infection present.

## Discussion

The prevalence of GIM found in this study among adults undergoing EGD at a private clinic in the Dominican Republic is consistent with previously reported global, North American, and Latin American prevalence rates.^1^^,^^9^ The antrum was identified as the most common lesion site, similar to findings from the U.S. and Latin American cohorts.^10^^,^^11^ Most cases in this study were of the complete subtype, differing from observations in Brazilian and U.S. studies, where incomplete GIM predominated.^9^^,^^12^

*H pylori* prevalence was lower than in reports from Dominican, Latin American, and Caribbean cohorts.^3^^,^^4^ Current and past *H pylori* infections and older age were independently associated with GIM risk after adjustment, consistent with findings of U.S. veteran populations.^13^ Although both increase risk, past *H pylori* infection showed a stronger association with extensive GIM, whereas current infection was more closely linked with limited GIM. This emphasizes that prior *H pylori* infection is a crucial risk factor for GIM and its progression.

The findings of this study provide essential data on GIM in the Dominican Republic by analyzing a large Dominican cohort and stratifying risk factors according to the extent of the lesions. The stronger association of past *H pylori* infection with extensive GIM demonstrates the long-term risks of this bacterium. Physicians and researchers do not always account for this factor. This shows its importance for patient management and future research analyses.

Male sex was more common among patients with GIM, which aligns with other studies.^13^ Although not significant, female sex appeared protective. Other established risk factors, such as family history of gastric cancer, alcohol and smoking history, GERD, dyspepsia, BMI, and use of PPIs and NSAIDs, were not significantly associated with GIM or its extension in this study, contrary to other research linking them with GIM presence and progression.^2^^,^^13^ This might be explained by the differences in population demographics and the existing data found in medical records. Because of the retrospective nature of the study, records often lacked detail on the quantity, frequency, and duration of exposures, which may have contributed to the lack of significance.

Screening for gastric cancer varies between Asia and the Americas. In Japan and Korea, national programs have improved early detection and reduced gastric cancer mortality.^14^^,^^15^ By contrast, in the United States, screening EGDs are not routinely performed on average-risk populations but are recommended for high-risk groups.^16^ Caribbean countries, particularly the Dominican Republic, lack systematic screening, resulting in GIM being detected only when endoscopy is clinically indicated. This may contribute to late-stage cancer diagnoses, an aspect to take into consideration, because gastric cancer rates in Caribbean populations are significantly higher than in France.^17^ Identifying risk factors in Dominican patients can be beneficial for potential future screening strategies.

This study had several limitations. The study was retrospective and conducted at a private clinic serving a higher-income demographic, which limited its generalizability to the Dominican population of other socioeconomic backgrounds. This selection bias could have explained the lower *H pylori* prevalence and the complete subtype predominance. Clinical data, including alcohol, smoking, use of PPIs, and NSAIDs, lacked detail regarding quantity and duration. Importantly, race and country of origin were not included in the patient's medical records. These are important risk factors for GIM that can be accounted for in future studies.

Because of the limited data that currently exist in regard to the risk factors associated with GIM in the Dominican Republic's general population, the findings of this study can only be compared with those from Dominicans living abroad, which may differ demographically. Future research is encouraged to build on these findings to clarify GIM's prevalence, its associated risk factors, and their potential relevance for developing gastric cancer screening strategies in the Dominican Republic, particularly among high-risk individuals. These studies could provide the evidence needed to support screening protocols, aiding in the earlier detection and prevention of advanced gastric lesions.

## Patient consent

Patient consent was obtained for the publication of this article.

## Disclosure

All authors disclosed no financial relationships.
